# Enzymatic Degradation of the Textile Dye Remazol Brilliant Blue R, a Model Recalcitrant Anthraquinone Dye

**DOI:** 10.3390/ijms27219982

**Published:** 2026-11-07

**Authors:** Pala Mahesh, Noel Biju Longhinos, Nagabalaji Velmurugan, Bipin Gopalakrishnan Nair, Sanjay Pal, Suja Subhash

**Affiliations:** 1 Amrita School of Biotechnology, Amrita Vishwa Vidyapeetham, Kollam 690525, Kerala, India; 2 Institute for Water and Wastewater Technology (IWWT), Durban University of Technology (DUT), Steve Biko Campus, Durban 4001, South Africa

**Keywords:** Remazol Brilliant Blue R (RBBR), anthraquinone dye, enzymatic degradation, textile water, bioremediation

## Abstract

The massive discharge of Remazol Brilliant Blue R (RBBR), a recalcitrant anthraquinone dye, is of great environmental and public health concern due to its persistence, toxicity, and resistance to conventional wastewater treatment technologies. Enzymatic biodegradation has been developed as a sustainable alternative due to its high substrate specificity, mild operating conditions, and reduced secondary pollution. The review critically analyses the catalytic mechanisms, degradation pathways, and bioremediation potential of laccases, peroxidases, and azoreductases in degrading RBBR. Unlike azo dyes, RBBR lacks an azo (–N=N–) linkage; therefore, oxidative enzymes, particularly laccases and peroxidases, constitute the primary catalytic systems responsible for anthraquinone chromophore oxidation, whereas the contribution of azoreductases appears to be complementary and dependent on enzyme specificity and microbial metabolic interactions. Recent experimental evidence obtained using UV–Vis spectroscopy, Fourier-transform infrared spectroscopy (FTIR), High-performance liquid chromatography (HPLC), Liquid chromatography–mass spectrometry (LC–MS), Gas chromatography–mass spectrometry (GC–MS), enzyme assays, and phytotoxicity analyses has considerably improved understanding of RBBR degradation mechanisms and intermediate metabolite formation. The review also compares the catalytic efficiencies, advantages, and limitations of the three enzyme systems. Recent advances in enzyme immobilization, protein engineering, microbial consortia, and hybrid biotechnological approaches to improve degradation efficiency and operational stability are discussed. Finally, future perspectives on omics-assisted enzyme discovery, advanced metabolomic characterization, pilot-scale validation, and integrated treatment technologies are outlined to facilitate the development of efficient, scalable, and environmentally sustainable strategies for the remediation of anthraquinone dye-contaminated wastewater.

## 1. Introduction

The textile industry is one of the largest manufacturing sectors worldwide and plays a vital role in the global economy. However, it is also among the most water- and chemical-intensive industries, consuming large quantities of dye and auxiliary chemicals during textile processing. Textile dyeing and finishing operations generate approximately 17–20% of global industrial wastewater, while 10–15% of synthetic dyes are lost during processing and discharged into aquatic environments, posing significant environmental and public health concerns [1]. The increasing demand for textiles has further intensified wastewater generation, making textile effluents a major source of environmental pollution [2].

Textile dyes are extensively used in industries such as textiles, paper, leather, food, cosmetics, and plastics due to their excellent color stability and durability [2]. However, synthetic dyes are designed to resist degradation by light, temperature, oxidizing agents, and microbial activity, allowing them to persist in the environment for prolonged periods [3]. Their discharge into aquatic ecosystems reduces light penetration, inhibits photosynthesis, decreases dissolved oxygen levels, and disrupts aquatic biodiversity. Moreover, textile dyes are recognized as persistent pollutants with toxic, mutagenic, and carcinogenic properties. Long-term exposure through inhalation, ingestion, or dermal contact has been associated with allergic reactions, respiratory disorders, and genotoxic effects, emphasizing the urgent need for efficient wastewater treatment technologies [2,4,5]. Various physicochemical dye-removal and treatment methods, including coagulation–flocculation, adsorption, membrane filtration, chemical oxidation, and advanced oxidation processes, have been employed for textile-wastewater treatment [4]. Coagulation–flocculation, adsorption, and membrane filtration primarily remove dyes through aggregation, phase transfer, retention, or separation rather than direct chemical destruction of dye molecules. Consequently, the contaminant may remain concentrated in sludge, spent adsorbents, or membrane retentates, creating disposal or regeneration challenges. In contrast, chemical oxidation and advanced oxidation processes can transform or degrade dye structures through oxidative bond cleavage; however, complete mineralization is not always achieved. Although these approaches can effectively reduce visible color, they may involve high operational costs, energy demand, chemical consumption, sludge generation, or formation of transformation products that require further assessment. Importantly, decolorization should not be equated with complete degradation or mineralization. Loss of color may result from alteration, cleavage, or transformation of the chromophoric structure while leaving residual organic fragments in the treated effluent. Therefore, treatment performance should be evaluated not only by color removal but also by chemical oxygen demand (COD) and total organic carbon (TOC) reduction, identification of transformation intermediates, and residual-toxicity assessment [5].

Biological treatment has emerged as an attractive alternative because of its cost-effectiveness and environmental compatibility. However, the degradation efficiency of whole microorganisms is frequently limited by dye toxicity, slow transformation rates, and sensitivity to environmental conditions. In addition, biological decolorization alone may not ensure detoxification or complete mineralization, particularly when persistent intermediate products accumulate. These limitations highlight the need for efficient biological approaches that promote deeper dye transformation while enabling assessment of treatment byproducts and residual toxicity. Enzyme-based treatment has gained particular attention because isolated biocatalysts can directly transform dye molecules without requiring maintenance of viable microbial cells. Oxidoreductases, including laccases, lignin peroxidases, manganese peroxidases, and azoreductases, can catalyze the cleavage or oxidation of chromophoric and aromatic structures under relatively mild reaction conditions. Among these enzymes, laccases are especially attractive because of their broad substrate range and ability to oxidize diverse phenolic and aromatic amine-containing compounds using molecular oxygen as the terminal electron acceptor, with water formed as the reduction product. Enzymatic systems may reduce dependence on harsh chemical oxidants and minimize secondary sludge generation; enzyme immobilization and redox-mediator systems can further improve enzyme stability, reusability, and oxidation of poorly accessible substrates. Nevertheless, the environmental performance of enzymatic treatment must be verified through analysis of transformation products, chemical oxygen demand (COD)/ total organic carbon (TOC) removal, and residual toxicity, as decolorization alone cannot confirm detoxification or complete mineralization [6,7].

Textile dyes undergo reductive and oxidative transformation via microbial enzymes, like laccases, peroxidases, and azoreductases, in a complementary fashion. Remazol Brilliant Blue R (RBBR) is a widely used model anthraquinone reactive dye with unique chemical, environmental, and treatment characteristics. Unlike azo dyes, RBBR degradation involves oxidative electron transfer and aromatic ring oxidation rather than azo-bond cleavage. Its high water solubility, chemical stability, and intense color make it resistant to conventional biological treatment and a suitable benchmark for enzymatic and advanced oxidation processes. The extensive literature on RBBR degradation, transformation products, and toxicity assessment provides sufficient material for a focused review. But their dominant enzymatic mechanisms to RBBR are dependent on dye chemical structure. Laccases and peroxidases are of special importance for RBBR, as their oxidative catalytic activities can directly initiate the transformation of the stable anthraquinone chromophore. Conversely, azoreductases are mainly related to the reductive cleavage of azo bonds (–N=N–) and therefore have a more limited and less documented direct role in RBBR degradation; their potential contribution possibly takes place through complementary reductive reactions within microbial consortia or sequential degradation pathways. This distinction is important in the interpretation of enzymatic degradation studies, as mechanisms established for azo dyes cannot be directly extrapolated to anthraquinone dyes such as RBBR. Although azoreductases do not directly cleave the anthraquinone structure of RBBR, they may contribute indirectly to dye degradation in mixed microbial consortia [8]. In textile effluents containing both azo and anthraquinone dyes, azoreductase-producing microorganisms can reduce azo bonds in co-existing dyes and transform certain redox-active intermediates [9]. This reductive activity may alter the redox environment, facilitate electron transfer, and generate intermediates that are more amenable to subsequent oxidative attack by laccases or peroxidases [10,11,12]. In sequential anaerobic–aerobic systems, azoreductase-mediated reduction may occur during the anaerobic phase, whereas oxidative enzymes can further transform residual chromophores and aromatic intermediates under aerobic conditions [13,14]. Therefore, the cooperation between azoreductases and oxidative enzymes in microbial consortia is likely to be complementary rather than direct: azoreductases may broaden the range of degradable dye components and support redox balancing, while laccases and peroxidases primarily initiate oxidative transformation and ring cleavage of RBBR [12]. Further studies using defined co-cultures, enzyme activity measurements, and intermediate-product identification are needed to confirm the extent and significance of this interaction.

**Figure 1:**
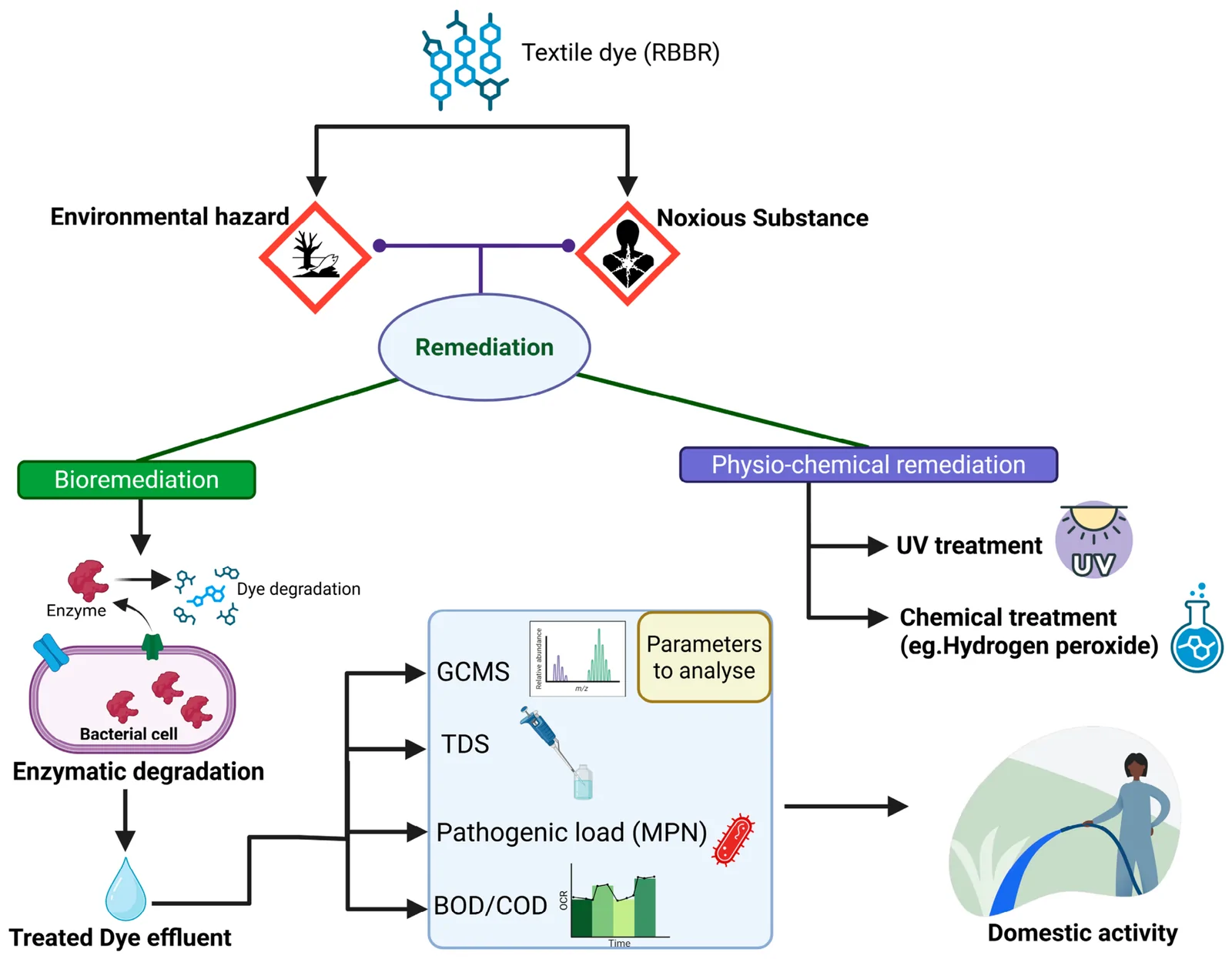
Schematic representation of the bioremediation process for toxic textile dye effluents [9]. Created in BioRender. Nair, B. (2026) https://BioRender.com/35bfryg.

RBBR is of particular interest because of its fused anthraquinone structure, which offers high chemical stability and recalcitrance; hence, it is a suitable model for the assessment of advanced enzymatic bioremediation strategies [15]. Although numerous studies have investigated microbial and enzymatic treatment of textile dyes, these approaches differ in their mode of action. Microbial degradation relies on intact living cells and may involve extracellular and intracellular enzyme-mediated transformation, cellular uptake, adsorption, and co-metabolic processes. In contrast, enzymatic degradation employs free, crude, partially purified, or immobilized enzymes to directly catalyze dye transformation without requiring viable microbial growth. Despite these advances, a comprehensive understanding of the enzymatic degradation mechanisms, catalytic pathways, and recent technological developments specifically associated with Remazol Brilliant Blue R (RBBR) remains limited. Therefore, this review provides an in-depth analysis of the enzymatic degradation of RBBR, with emphasis on the enzymes involved, catalytic mechanisms, influencing factors, recent advances, current challenges, and prospects for sustainable textile-wastewater treatment. The bioremediation process involved in the degradation of toxic textile dyes is schematically illustrated in Figure 1.

## 2. Classification of Dyes

Dyes are organic substances that confer color to natural and synthetic materials. These are extensively used in the textile, paper, leather, food, and cosmetic industries [16]. Most dyes consist of aromatic structures and chromophoric groups that provide color and excellent chemical stability. Dyes are extensively used in various industries, and their ionization ability, in most cases, and easy solubility in water aid their distribution in the environment. Thus, dye-contaminated wastewater poses a serious environmental problem [17]. Dyes are classified into three classes based on their ionic nature: they are anionic, cationic, and nonionic. Anionic dyes are reactive, acidic, and direct dyes with negatively charged sulfonate or carboxylate groups. Cationic or basic dyes have positively charged quaternary ammonium groups. Nonionic dyes, like disperse dyes, for instance, show a low solubility in water and are mainly used for synthetic fibers [16].

**Figure 2:**
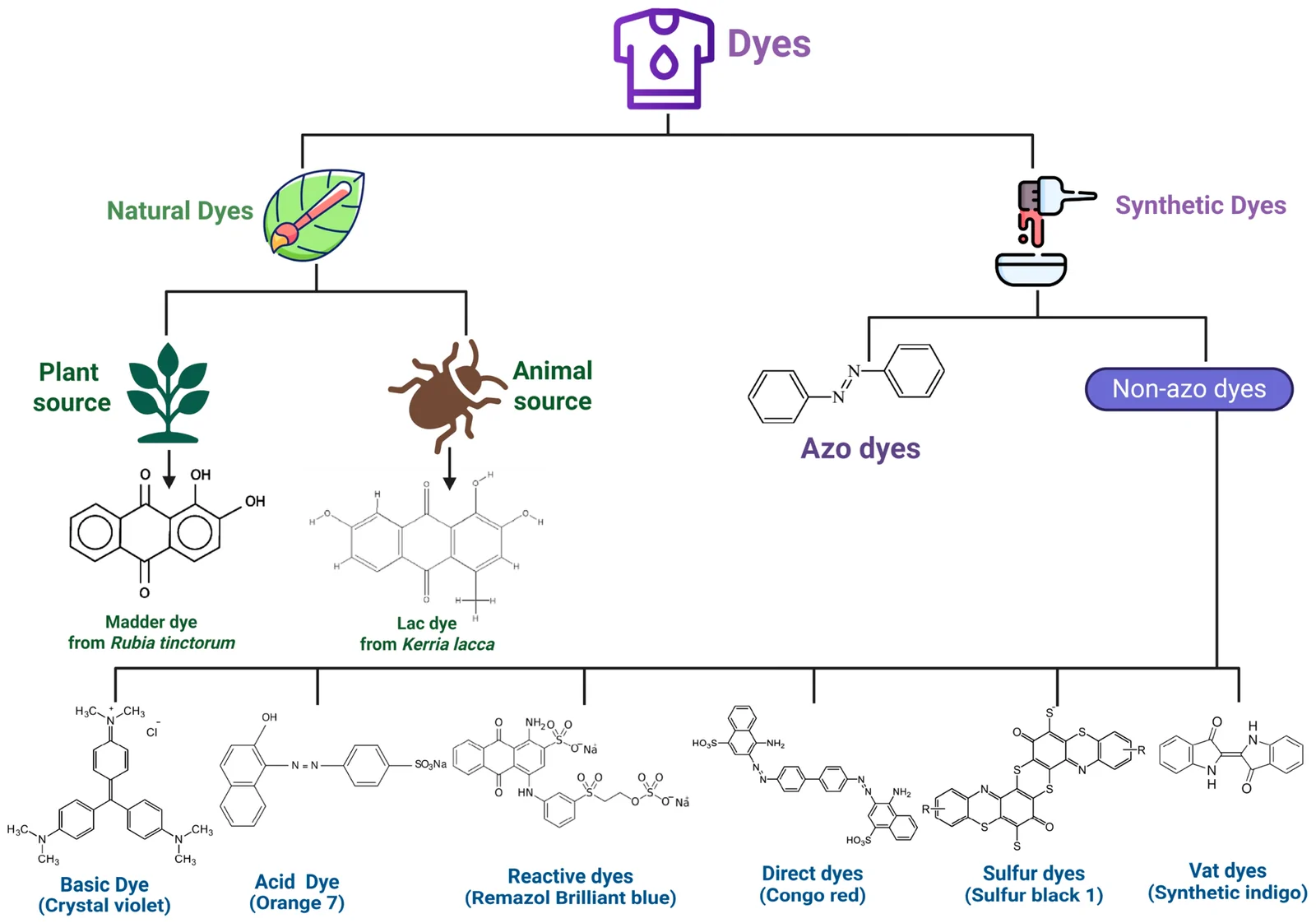
Examples of popular textile dyes based on their origin and mode of application [15,20,21]. Created in BioRender. Pal, D. S. (2026) https://BioRender.com/eeehly0.

Dyes are also classified by chemical structures such as azo, anthraquinone, phthalocyanine, triphenylmethane, sulfur, indigoid, nitro, nitroso, and metal-complex dyes (Figure 2). Among these, azo dyes are the most abundant, accounting for nearly 60–70% of the dyes produced commercially. Anthraquinone dyes represent the second largest class of dyes and are extensively used owing to their brilliant colorization, excellent light fastness, and high resistance towards chemical and biological degradation [17]. These dyes are characterized by an anthraquinone chromophore comprising fused aromatic rings with two carbonyl (quinone) groups, which impart extraordinary structural stability, and thus they are very persistent in aquatic media and resistant to removal by conventional treatment methods.

These dyes have a wide range of structural stability and susceptibility to enzymatic transformation. Anthraquinone dyes are characterized by an aromatic framework with a fused quinone-based structure, conferring greater structural stability and resistance as compared to azo dyes, which generally undergo transformation by azoreductases and contain one or more azo (–N=N–) linkages [1]. RBBR is an anthraquinone dye, and this structural class is particularly relevant to enzymatic degradation because its color arises from a highly conjugated quinone chromophore. Although this chromophore contributes to chemical stability and resistance to conventional treatment, it can be selectively oxidized by enzymes such as laccases and peroxidases. These enzymes initiate one-electron oxidation or radical-mediated reactions that alter or cleave the anthraquinone structure, disrupting the conjugated system and causing decolorization. Subsequent oxidation may further convert the resulting intermediates into lower-molecular-weight products, although complete mineralization depends on the enzyme system and reaction conditions [18,19].

### Recalcitrance of Remazol Brilliant Blue (RBBR)

RBBR is very resistant to the normal biological treatment systems, especially to the aerobic activated sludge processes. The resistance of the compound to biological degradation is mainly attributed to the fused aromatic anthraquinone skeleton, the strong electronic deactivation by electronegative substituents, the high hydrophilicity preventing passive transmembrane transport, and the steric barriers limiting active-site binding in non-specialized microbial enzymes [22,23,24]. Extensive electron delocalization arises from the two planar benzene rings fused to a central para-quinoid ring. This cyclic conjugated resonance minimizes the free energy of the molecule and provides it with exceptional thermodynamic stability. Therefore, the aromatic core is highly resistant to non-specific enzymatic decomposition and nucleophilic and electrophilic attack in normal biological treatment conditions [24,25]. The resistance of the anthraquinone nucleus is further enhanced by the peripheral substituents of the dye: an electron-withdrawing sulfatoethylsulfone (SO_2_-CH_2_-CH_2_-OSO_3_Na) functional group, which upon dyeing converts into an active vinyl sulfone (SO_2_-CH=CH_2_) crosslinker [25]. An aryl sulfonic acid group (SO_3_Na) directly attaches to the anthraquinone core. These electronegative moieties exhibit strong electron-withdrawing inductive and resonance effects, leading to a decrease in the electron density in the whole aromatic framework [26]. Common bacterial catabolic oxygenases and oxidases initiate cleavage by electrophilic aromatic oxidation, and thus electron-deficient rings pose an energetic barrier to catalysis [26]. In addition, molecular docking studies indicate that the bulky sulfatoethylsulfone and polar sulfonate side chains lead to steric hindrance and therefore perfect conformational fits, and specific catalytic architectures (e.g., special multicopper oxidases or laccases) are required to place the substrate into enzymatic binding cavities [26,27].

Moreover, RBBR is anionic and highly water-soluble due to the presence of several charged, water-solubilizing sulfonate groups. The usual bacterial biodegradation proceeds through intracellular assimilation followed by catabolic degradation. Nevertheless, the polyanionic nature, high polarity, and molecular weight (626.54 g/mol) of RBBR prevent passive permeation through the hydrophobic lipopolysaccharide and lipid bilayer matrices of bacterial cell walls [28]. The dye is retained in the extracellular phase, as the conventional wastewater microorganisms do not possess transport systems for synthetic anthraquinones [28].

RBBR has resistance to solar radiation, perspiration, laundering, and chemical oxidation. There are no structural analogs in natural biogeochemical cycles. Conventional activated sludge biota have not evolved catabolic cascades for such xenobiotics [24,27]. Degradation often requires specialized extracellular oxidoreductases, such as the ligninolytic enzymes from white-rot fungi or robust bacterial laccases (e.g., *Geobacillus* spp.) that can perform non-specific single-electron oxidations outside the cell, systems that are missing in standard biological wastewater plants [26,28].

## 3. Effects of Textile Dyes on the Environment

The textile industry generates large amounts of untreated wastewater containing diverse organic pollutants, including azo dyes and persistent anthraquinone compounds, such as RBBR [29]. When they are released, these pigments degrade water quality, odor, and aesthetics substantially. They also block sunlight from entering aquatic environments. This bright barrier limits basic aquatic productivity and prevents the photosynthesis of aquatic vegetation. Dyes are partially degraded before or after disposal, but their degradation forms xenobiotic metabolites. Dangerous aromatic intermediates/quinones from RBBR ring-opening and aromatic amines from azo dye cleavage are often much more hazardous than the original compounds [30]. Mammals are mainly exposed through two ways, ingestion and skin contact [31]. Upon ingestion, the intestinal microbiota converts these initial colors and intermediates into toxic aromatic amines that may induce cytotoxic effects, severe skin irritations, allergic reactions, and increased risk of cancer in host tissues.

### 3.1. Effects of Textile Dyes on Human Health

Fish and other aquatic animals are important sources of protein for humans, and they easily absorb pigments from the environment. Textile effluents are characterized by brilliant colors, variable pH, and complex mixtures of salts and acids that elevate biological oxygen demand (BOD), chemical oxygen demand (COD), and suspended solids [32]. Suspended solids can slow growth and limit gas exchange by blocking water flow across fish gills. Reactive dyes cause blood disorders, including the formation of erythrocytic micronuclei. Moreover, hypoxia tremendously reduces immune efficiency, resulting in increased health risks for human consumers.

Anthropogenic dyes have been strongly linked to systemic toxicities, dermatitis, and central nervous system complications, mainly through ingestion, inhalation, or dermal contact [33]. Well-established mutagens, e.g., Disperse Red 1 [34], and azo-derived aromatic amines (e.g., 1,4-diaminobenzene), cause severe contact dermatitis, chemosis, lacrimation, and respiratory distress.

Compared to the amino-cleavage toxicity of some azo dyes, the structural stability of the fused anthraquinone framework determines the environmental persistence and biological interactions of anthraquinone dyes such as RBBR. Rybczyńska-Tkaczyk et al. 2026 [35] reported that RBBR upregulates CYP1A2 and CYP2B6 mRNA expression in HepG2 cells. In yeast-based reporter assays, RBBR exhibited estrogenic activity at concentrations of 3–100 uM and androgen-receptor agonistic activity at concentrations of 30–100 uM, indicating potential endocrine-disrupting activity at concentration-dependent levels. Toxicological effects of RBBR are model- and exposure-dependent, but these results provide experimental evidence for possible interactions of RBBR with cellular metabolic and endocrine-related pathways. As the successful enzymatic transformation of the parent dye and possibly harmful intermediates is crucial for real detoxification, the assessment of the enzymatic degradation of RBBR should not only consider the decolorization efficiency but also the reduction in biological toxicity [35].

### 3.2. Effect of Textile Dyes on Plants

Dyes pose significant health and environmental concerns due to their diverse hazardous effects [36]. The toxicity of an environment is dependent on the amount and distribution of each toxic element present and, in turn, affects the diversity of the ecosystem. Phytotoxicity bioassays are useful tools for testing the toxicity of synthetic dyes and controlling pollution in the environment. The amount of dye in the water, the amount of metal in the soil, the solubility level, and the type of plant are some of the factors that influence the absorption and accumulation of dye and minerals in crops and other plants. Leafy vegetables have a greater potential to take up and accumulate metals because they transpire and translocate more than other plant species. Heavy metals can thus rapidly be moved from the roots to the leaves of leafy greens. On the other hand, the rate of metal transfer from roots to the stem and fruits is slower in non-leafy vegetables; thus, lower metal accumulation levels are obtained [37].

RBBR results in serious phytotoxic effects by damaging crucial physiological and developmental stages in plants [37]. The anthraquinone structure and the reactive vinyl sulfone groups of the compound are stable and inhibit the seed germination indices and significantly reduce root and shoot elongation, such as radicle and plumule growth [38]. RBBR uptake by cells is linked to oxidative stress, leading to cell membrane damage and inhibition of photosynthetic pigment production [37]. This impacts the whole plant metabolism and biomass accumulation [38]. However, enzymatic or microbial degradation, such as laccase-producing bioinoculants, is efficient at degrading the complex aromatic rings, which greatly decreases the phytotoxicity and restores the plant growth potential [37].

In addition to dye removal, detoxification should also be considered because exposure to RBBR has been correlated to measurable phytotoxic effects. The experiments showed that enzymatic degradation could significantly reduce the biological toxicity of RBBR. Furthermore, *Arthrographis kalrae* produced laccase during RBBR degradation, and it significantly reduced the phyto- and cytotoxicity of the dye in comparison to RBBR along with high decolorization efficiency [22]. Verma and Shirkot (2018) demonstrated the reduced phytotoxicity of the transformation products of RBBR formed by bacterial laccase in experiments on the seed germination of *Phaseolus vulgaris* [39]. The breakdown of RBBR by *Staphylococcus* spp. K2204 was caused by laccase and peroxidase enzymes that were released from the cells. The degradation products were identified by mass spectrometry, and their phytotoxicity was evaluated to estimate the detoxification capacity of the treatment [40]. Collectively, these results indicate that RBBR can be decolorized and biological toxicity can be reduced by enzymatic treatment. Therefore, the assessment of phytotoxicity is an important endpoint for evaluating the effectiveness of enzymatic remediation [39,40].

### 3.3. Effect of Textile Dyes on Water

Textile dyeing effluents are one of the major pollutants of concern and pose a serious threat to aquatic ecosystems. On the contrary, potable water is mainly obtained from groundwater, which is not contaminated and plays an important role in preserving water quality [41]. The volume of wastewater produced by the regional textile and garment industry is astonishing. RBBR contamination can impair water quality by persistent colorization and reduced light penetration, potentially disturbing photosynthetic activity in aquatic ecosystems. Its recalcitration anthraquinone structure and toxic effects may adversely affect aquatic microorganisms; it is essential to minimize its ecotoxicology impact on receiving water bodies [35].

Two of the largest producers of textiles, India and China, face enormous problems in the treatment of textile wastewater containing dyes. For example, in the Tiruppur textile cluster (India), 729 dyeing units discharge almost 96.1 MLD (million liters per day) of wastewater with high total dissolved solids (TDS) and chloride levels affecting the Noyyal River [42]. In China, a pilot-scale textile-wastewater reclamation system (600 m^3^/day) with biological treatment and membrane technology achieved about 93% COD and 94.5% color removal, indicating the potential for reuse of wastewater in textile processing [43]. The above cases reveal the levels of textile-wastewater pollution and the importance of sustainable treatment and reuse approaches in the countries producing the major textile products. Similarly Bangladesh’s industry produced around 217 million cubic meters of wastewater in 2016; this number is projected to exceed 349 million cubic meters recently, considering that processing a kilogram of fabric can require up to 150 L of water [44,45]. Although many textile dyeing industries are currently in operation, historical estimates show that more than 80% of them lack the technological infrastructure to treat wastewater, and in combining units with knitting, dyeing, and finishing operations, only 2% of the units have fully operational treatment plants [45]. Regional statistics indicate that approximately 61% of the wet processing facilities are reported to have effluent treatment plants (ETPs), of which only 29% are functionally compliant with environmental discharge standards, whereas 11–51% are poorly designed or non-operational, even with recent infrastructural developments [45].

Most studies on RBBR degradation have been performed using pure or simulated dye solutions, which may not represent the complexity of real textile effluents [46]. Real effluents commonly contain high concentrations of salts, surfactants, auxiliary chemicals, heavy metals, and mixtures of dyes, all of which can affect enzyme activity, stability, and substrate accessibility. High ionic strength from salts such as sodium chloride and sodium sulfate can alter enzyme hydration and conformation, interfere with substrate binding or cofactor/mediator interactions, and reduce catalytic efficiency or operational stability. For example, 80 mM NaCl reduced the activity of *Mycecliophthora thermophila* and *Trametes trogii* laccases by 37% and 45%, respectively, at pH 4.5 [47]. Surfactants may denature enzymes, modify enzyme–substrate interactions, or alter dye solubility and availability; their effects depend strongly on surfactant type, concentration, and exposure time [46,47]. Auxiliary dyes and finishing chemicals may compete with RBBR for enzyme active sites, consume redox mediators or oxidants, produce inhibitory intermediates, or interfere with color-based analytical measurements [48]. Heavy metals can also bind to catalytic sites or redox-active cofactors, leading to reversible or irreversible enzyme inhibition. Therefore, the practical applicability of enzymatic RBBR treatment should be evaluated using real or matrix-matched textile effluents, with assessment of residual enzyme activity, decolorization efficiency, enzyme stability, degradation products, and toxicity [49].

### 3.4. Effect of Textile Dyes on Soil

Soil is a living ecosystem of minerals and organic matter. It is the basis for agricultural production and plant growth. The dumping of textile effluents in uncontrolled ways on agricultural land alters the pore structure and increases the contaminant loading and nutrient availability, which leads to a decrease in soil quality [50].

RBBR contamination can adversely affect the soil–plant systems by reducing seed germination and root growth, with phytotoxic effects becoming more pronounced at increasing the dye concentration [51,52]. The persistence of RBBR in the soil environment may impair plant establishment and limit the safe agricultural reuse of contaminated sites. Biological degradation of RBBR could therefore provide a sustainable approach for reducing its phytotoxicity and improving the environmental quality of contaminated soil. Some experiments have shown that dilution of textile effluent is a beneficial condition for irrigation [53] but is not applicable in all cases. The appropriate dilution ratio depends on the physicochemical composition of the wastewater as well as the characteristics of the receiving soil, such as salinity, pH, nutrient status, texture, organic matter content, and drainage capacity. Naseem et al. (2022) [53] demonstrated that a 25% dilution of textile wastewater when paired with plant growth-promoting bioinoculants like *Enterobacter cloacae* ZA14, can successfully supply essential nutrients and enhance seedling vigor, controlled effluent reuse presenting a promising avenue for water conservation in agriculture; however, because raw industrial discharges vary greatly in composition and require careful management to prevent environmental imbalances, a diverse suite of advanced physical, chemical, biological, and enzymatic cleaning technologies remains essential to reliably pretreat wastewater, ensure optimal water quality, and safely unlock its full reuse potential across varied agricultural settings. Therefore, a characterization of wastewater and a site-specific soil assessment are required to determine a safe and agronomically suitable dilution ratio before irrigation. Textile effluents may adversely affect seed germination even at relatively low concentrations, and the long-term application may increase the accumulation of salt and other pollutants in soil. Hence, adequate treatment, dilution, and monitoring are necessary to reduce the risks involved with the reuse of textile effluent in agriculture [52].

## 4. Physical and Chemical Methods of Wastewater Treatment

Wastewater from textile processing is treated using physical, chemical, and biological methods [54]. Biological approaches historically rely on whole-organism systems, including phytoremediation, activated lagoons, and trickling filters [55]. However, phytoremediation faces operational challenges like long treatment cycles, large land footprints, and seasonal instability [56]. Consequently, attention has shifted toward microbial strategies, employing microbes as free, immobilized, or desiccated biomass [54].

Despite their efficiency, intact microbial systems struggle in real effluents due to dye toxicity, extreme pH, and high salinity, which cause cell inactivation [56]. Moreover, whole cells often rely on physical biosorption rather than true degradation, leaving intact dyes bound to sludge [57].

To overcome these limitations, research has increasingly focused on enzymatic degradation as a targeted, cell-free alternative. Instead of depending on microbial survival, extracellular enzymes like laccases, peroxidases, and azoreductases directly cleave specific bonds within recalcitrant chromophores [31]. This transitions wastewater remediation from simple physical accumulation to destructive catalytic mineralization, efficiently converting complex dye structures into less toxic, lower-molecular-weight compounds under mild operational conditions [31]. For RBBR specifically, physicochemical methods such as adsorption, membrane separation, coagulation–flocculation, electrochemical treatment, and advanced oxidation processes have been investigated. These methods can achieve substantial decolorization, but adsorption and coagulation mainly transfer the dye to another phase, whereas membrane systems generate concentrated streams and oxidation-based approaches may produce incompletely characterized intermediates. Therefore, enzymatic degradation offers a complementary strategy that can directly transform the RBBR chromophore under milder conditions (Table 1).

### 4.1. Recent Trends in Physical Methods

Decolorization of wastewater containing dispersion dyes and other substances can also be achieved by physical methods. However, these methods have limitations in their usage due to limited effectiveness in color removal and generation of huge amounts of sludge. To make adsorption more affordable, some researchers have investigated cost-effective adsorption materials such as peat, bentonite clay, fly ash, and polymeric resins [70,71]. Nevertheless, there are numerous challenges associated with these adsorbents. Therefore, it is advisable to employ adsorbents in scenarios where pollutant concentrations are low or when the adsorbent is inexpensive and can be easily regenerated [72]. The subsequent section will examine various physical techniques applicable for water treatment. Some of the advanced methods are as follows:

Physical separation techniques such as microfiltration (MF), nanofiltration (NF), and reverse osmosis (RO) membrane-filtration technologies are widely used in the treatment of textile effluents. Microfiltration is a low-pressure process using membranes with pore sizes in the range of 0.1 and 1.0 μm, it is mainly used as a pretreatment step before high-pressure filtration. While MF effectively removes suspended solids, colloidal particles, and large dye aggregates, it is unable to retain dissolved low-molecular-weight dye molecules due to its pore size [73]. In contrast, nanofiltration has a higher hydraulic permeability and a lower transmembrane pressure than conventional reverse osmosis with a pore size of 1–2 nm. As a result, NF has been an appropriate technique for the elimination of multi-charged reactive dyes with less power consumption in operation [74]. Reverse osmosis is an advanced polishing step that retains 90–99% of ionic species, hydrolysed reactive dyes, and low-molecular-weight chemical complexes, resulting in a permeate suitable for direct recycling in the plant [75].

Membrane-based separation and adsorption using biochar are two sustainable and promising methods for the removal of textile dyes. Biochar is a product of agricultural and other biomass residues. It has a large surface area, a porous structure, and a series of surface functional groups. These properties make it very suitable for adsorption of dyes through electrostatic interactions, π-π interactions, hydrogen bonding, and pore filling. This feedstock is low cost, renewable, and has the potential for regeneration; hence, it is suitable for the treatment of wastewater, as reported by Saratale et al. (2022) [26] and Y. Wang et al. (2024) [76]. Adsorption can transfer dye molecules from the aqueous phase to the solid phase but does not necessarily achieve full degradation or detoxification of the chemicals. Recent studies have focused on biochar-enhanced biological treatments to improve the dye removal by the synergistic effect of biochar’s adsorption and redox properties and microbial degradation [55]. Hence, the co-use of biochar-based adsorption and enzymatic treatment can be applied for RBBR remediation with efficient pollutant trapping and further enzymatic conversion and purification.

Despite high initial color removal efficiencies exceeding 95% for reactive anthraquinone dyes such as Remazol Brilliant Blue R (RBBR) driven by their relatively high molecular weight and anionic sulfonate groups, physical membrane separation presents fundamental operational limitations. Foremost among these is the non-destructive nature of physical separation; these systems do not cleave or mineralize the recalcitrant, fused 9,10-anthracenedione core of RBBR. Conversely, they only concentrate the dye into a high concentration retentate stream in the bulk effluent, generating a secondary hazardous sludge with complicated disposal methods or requiring secondary treatment [55].

### 4.2. Biological Methods

The methods of biological treatment of dyes use live or inactive microbial biomass or enzymes to degrade the dyes and offer several advantages over physico-chemical methods. These techniques are mainly based on microbial adsorption or enzymatic degradation, which makes the application easier, with less chemical use, minimum sludge production, and low energy consumption. Also, bioremediation byproducts are generally less toxic. The major aim is the total transformation of the toxic organic dyes to simpler non-toxic inorganic substances [77]. While most of the dye bioremediation literature focuses on azo dyes, the degradation of anthraquinone dyes such as Remazol Brilliant Blue R (RBBR) follows a different pathway owing to their complex fused aromatic ring structure [41]. Biological degradation of RBBR and other anthraquinone dyes usually occurs through the enzymatic action of oxidative enzymes such as laccase, lignin peroxidase (LiP), and manganese peroxidase (MnP), which are mainly secreted by some bacteria and white-rot fungi [27].

The enzymes, laccase, lignin peroxidase (LiP), and manganese peroxidase (MnP) are the primary catalytic components of the extracellular ligninolytic system, predominantly secreted by white-rot fungi and certain bacteria to depolymerize complex, insoluble matrices in nature [78]. Structurally, laccases are multicopper oxidases containing four tightly bound copper ions (classified as Type 1, Type 2, and a binuclear Type 3 cluster) at their active site. This configuration allows laccases to couple the oxidation of a broad range of substrates directly to the four-electron reduction of molecular oxygen (O_2_) to water [79]. In contrast, both LiP and MnP are heme-containing peroxidases. Their structural core relies on an iron-protoporphyrin IX catalytic center that strictly requires hydrogen peroxide (H_2_O_2_) as an electron acceptor to activate their catalytic cycle [80]. While LiP possesses a remarkably high redox potential capable of directly oxidizing non-phenolic aromatic compounds, MnP functions by oxidizing Mn^2+^ to highly reactive Mn^3+^, which then acts as a diffusible redox mediator to attack complex substrates [81]. Despite these structural differences in their catalytic centers and co-substrate requirements (O_2_ vs. H_2_O_2_), they share a fundamental, overarching functional mechanism: the ability to catalyze the non-specific, one-electron oxidation of substrates. This shared radical-mediated mechanism generates highly unstable reactive intermediates (such as cation and phenoxy radicals) that undergo spontaneous, non-enzymatic cleavage reactions [82]. Because they rely on this radical generation rather than a strict lock-and-key active-site binding mechanism, these enzymes exhibit extraordinarily broad substrate specificity. This shared mechanism makes the ligninolytic enzyme consortium exceptionally capable of degrading a vast spectrum of complex, recalcitrant aromatic pollutants, ranging from synthetic dyes to pharmaceutical compounds in wastewater [78,82].

**Scheme 1:**
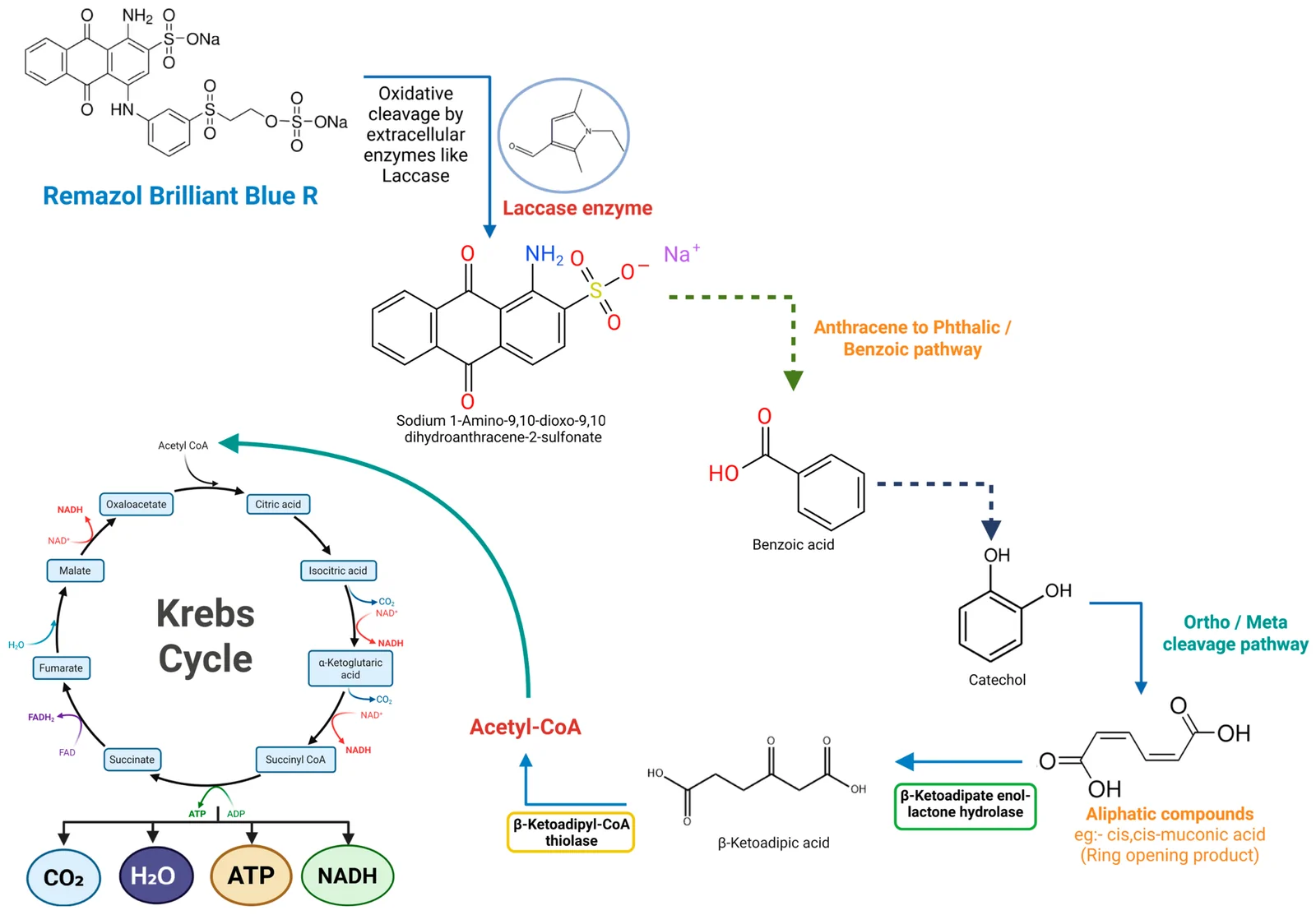
Proposed biological degradation pathway of the anthraquinone dye Remazol Brilliant Blue R (RBBR) [83,84,85]. Created in BioRender. Pal, D. S. (2026) https://BioRender.com/0uz9btd.

The first oxidation in the RBBR transformation can lead to the formation of reactive intermediates and destruction of the chromophore in the conjugated anthraquinone system. Smaller intermediates are formed by subsequent oxidative cleavage and transformation of aromatic structures, followed by additional ring cleavage/opening and, when shown, mineralization. Decolorization is therefore mainly the disruption of the chromophore, and ring opening is a secondary structural change and not just the loss of color [34]. The biological degradation, pathway of anthraquinone dyes usually begins with enzymatic oxidation of the dye molecule, which leads to the degradation of the complex anthraquinone ring [83]. Oxidative enzymes owe this activity to specific redox-active cofactors: laccases contain multicopper centers that mediate one-electron oxidation of phenolic and aromatic substrates with the reduction of oxygen to water, whereas peroxidases contain heme groups and use hydrogen peroxide to form high-valent iron-oxo intermediates capable of oxidizing electron-rich aromatic structures. This key ring-opening step destroys the chromophore, resulting in rapid visual decolorization. Mechanistically, initial oxidation generates radical, quinone, or quinone-imine intermediates, which may undergo hydroxylation and oxidative cleavage of adjacent carbon–carbon bonds. These ring-opening reactions disrupt the conjugated aromatic system responsible for color and produce lower-molecular-weight intermediates. After the first breakdown, intermediate substances are created, mainly phthalic acid and its related compounds [83,84,85,86]. These smaller aromatic intermediates are further degraded under aerobic conditions, entering the central metabolic pathways (e.g., the tricarboxylic acid cycle (TCA) cycle), where they are ultimately mineralized to carbon dioxide, water, and biomass. Scheme 1 is a proposed biological degradation pathway of the anthraquinone dye Remazol Brilliant Blue R (RBBR).

Microorganisms have the potential to interact and transform the aromatic compounds through several mechanisms, such as biosorption and intracellular and extracellular enzymatic activity via metabolic processes. However, the predominant mechanisms vary among microbial groups. This process occurs under both aerobic and anaerobic conditions. Aerobic conditions refer to conditions in which molecular oxygen (O_2_) is available to support microbial respiration and oxidative metabolism. This indicates oxygen availability facilitates subsequent oxidative transformation of aromatic cleavage products. However, the complete mineralization of the cleavage products of the anthraquinone ring generally requires sequential aerobic and anaerobic processes. This bioremediation process is performed by microorganisms such as bacteria, algae, filamentous fungi, and yeast. Recently, the biological treatment of RBBR has focused on optimizing environmental conditions for novel microbial consortia, using genetically modified strains for hyper-production of enzymes, and immobilizing biomass to improve the reusability and efficiency of the degradation process [87].

Kinetic studies provide useful information for comparing the catalytic performance of different laccase systems during RBBR degradation. For example, laccase from *Coriolopsis caperata* followed Michaelis–Menten kinetics for RBBR decolorization, with an apparent $K_{m}$ of 1.06 mM, $V_{max}$ of 0.226 mM U^−1^ min^−1^, $k_{cat}$ of 135 s^−1^, and catalytic efficiency $\left(k_{cat}/K_{m}\right)$ of $1.27\times 10^{5}$ s^−1^ M^−1^ [86]. In another study, *Pleurotus florida* laccase exhibited $K_{m}$ and $V_{max}$ values of 145.82 mg L^−1^ and 24.86 mg L^−1^ min^−1^, respectively, for RBBR decolorization [87]. The kinetic behavior of laccase-mediated RBBR removal has also been evaluated for free and immobilized enzymes: laccase from *Oudemansiella canarii* showed $K_{m}$ values of 0.126 and 0.159 mmol L^−1^ and $V_{max}$ values of 1.412 and 1.214 µmol min^−1^ in free and immobilized forms, respectively [88]. These results indicate that enzyme source, immobilization, dye concentration, pH, temperature, and the presence of mediators can substantially influence the apparent kinetic parameters. Therefore, kinetic data should be compared under comparable experimental conditions and interpreted together with decolorization efficiency, enzyme stability, and degradation-product analysis.

#### 4.2.1. Bacterial Systems for Dye Decolorization and RBBR Degradation

Bacterial strains offer a convenient and environmentally sustainable approach for the degradation and removal of dye compounds. These microorganisms possess enzymatic systems that enable efficient dye degradation or biosorption [89]. Bacteria have advantageous characteristics such as a shorter life cycle and the capability to break down and convert dyes into minerals [90]. Numerous research endeavors have employed bacterial strains to degrade and eliminate diverse textile wastewater containing azo dyes. Instances encompass microorganisms (attaining a 95% reduction in color), reactive yellow (84%), and reactive black 5 (complete eradication). Furthermore, these strains exhibit proficient color elimination capabilities, when addressing anthraquinone-based dyes such as Remazol Brilliant Blue (with a decolorization rate of 93.3) [91].

However, the degradation of anthraquinone dye RBBR is mainly through oxidative pathways because of its stable fused aromatic ring structure, which is different from azo and other typical textile dyes that are mainly degraded by reductive cleavage of azo bonds. Also, some bacterial taxa such as *Staphylococcus* spp., *Bacillus* spp. and *Pseudomonas* spp. have been reported to effectively attack this recalcitrant structure [39,40,92]. The degradation is mainly facilitated by extracellular and cell-bound oxidoreductases, particularly laccases and dye-deconvoluting peroxidases [93]. Laccases catalyze the one-electron oxidation of the anthraquinone nucleus with the formation of radical intermediates, resulting in ring cleavage and detoxification [34]. The emphasis on these enzymatic mechanisms explains the unique ability of these selected bacterial strains to transform RBBR into lower-molecular-weight and less toxic intermediates, rather than the general and nonspecific microbial decolorization [92].

#### 4.2.2. Fungal-Mediated Degradation of Remazol Brilliant Blue R (RBBR)

Fungal mycelia have a physiological advantage over unicellular microorganisms because of their hyphal structure that allows the degradation of insoluble compounds and secretion of extracellular enzymatic catalysts [94]. The structural morphology results in a very high surface area to volume ratio, enhancing the physical and enzymatic interactions with the surrounding microenvironment. Thus, many fungal species such as *Aspergillus niger*, strains of *Rhizopus*, and white-rot fungi have shown excellent potential for decolorization and degradation of a wide range of synthetic dyes [95]. White-rot fungi (WRF) have been reported surprisingly efficient among biological agents, especially in the degradation of the refractory anthraquinone dye Remazol Brilliant Blue R (RBBR). The better performance of these mutants is due to the presence of strong non-specific extracellular lignolytic systems formed by laccases, manganese peroxidases (MnP), and lignin peroxidases (LiP) with high redox potentials, able to cleave fused aromatic ring structures [96]. Important WRF species such as *Trametes versicolor*, *Pleurotus ostreatus*, *Phanerochaete chrysosporium* and *Trametes hirsuta* usually achieve nearly complete RBBR removal (>90%) which is considerably higher than single-celled bacterial systems due to the synergistic effect of extensive mycelial biosorption and targeted oxidative enzymatic degradation [96,97].

White-rot fungi can degrade textile dyes due to their extracellular ligninolytic enzyme systems, namely laccases, manganese peroxidases, and lignin peroxidases. *Trametes versicolor* is the most studied fungal species and decolorizes structurally diverse textile dyes, including anthraquinone dyes such as RBBR. Experiments show that the crude enzymes of *T. versicolor* decolorized RBBR at 100 mg L^−1^ in 24 h, revealing the extracellular oxidative enzyme system of *T. versicolor* [98]. Laccase from *T. versicolor* could decolorize RBBR by 93% in 3 h without the presence of any external mediator, indicating its strong oxidative capacity toward anthraquinone dyes. Recently, laccase from *T. versicolor* has shown remarkable activity for decolorization of textile dyes, thereby improving the fungal oxidative enzymes used for dye remediation [99].

#### 4.2.3. Algal Systems for RBBR Biosorption and Enzymatic Transformation

Algae are advantageous because of their several metabolic pathways and special oxidoreductase enzymes that can detoxify the synthetic colors found in the textile effluent [79]. Microalgae decolorize dyes by a joint process of physical biosorption, bioaccumulation, and enzymatic biodegradation [58]. In removing complex aromatic compounds from water (e.g., Congo Red, Brilliant Blue R, and the refractory anthraquinone dye Remazol Brilliant Blue R (RBBR), *Chlorella vulgaris* and *Scenedesmus* strains have demonstrated high decolorization efficiencies (>85 to 90%) [58,100].

The decolorization efficiency should be differentiated because the removal of RBBR in microalgal systems could be attributed to biosorption or enzymatic degradation. Biosorption is the binding of RBBR to the functional groups present on the surface of the algal cells. Enzymatic degradation is a biochemical transformation of the structure of the dye. Biosorption has been useful in the removal of dyes. *Scenedesmus quadricauda* showed efficient adsorption of RBBR [101]. Microalgae and *cyanobacteria* can synthesize enzymes that can degrade pollutants. These can be either extracellular or cell-associated. The loss in concentration of RBBR or color is not necessarily indicative of enzymatic degradation. Any dye associated with biomass, enzyme activity, and degradation products should be determined. Recent work on RBBR indicates concurrent biosorption and enzymatic degradation, with enzymatic transformation prevailing at optimal conditions [77].

In algae, degradation of RBBR and other polycyclic dyes is specific and is performed mostly by internal and extracellular enzymes, mainly peroxidases, laccases, and reductases [100]. These algae enzymes are specific to the fused stable aromatic rings of anthraquinone molecules. Extracellular peroxidases can catalytically perform single-electron oxidation reactions to cleave the ring and degrade the toxic parent molecule to smaller metabolites, which can be eliminated [102]. This process gives rise to reactive radical intermediates. The active bioaccumulation and cellular sequestration through functional groups present in the cellular matrix can retain the dye molecules in the algal cells [58]. This supports a long interaction time of enzymes and efficient detoxification of textile effluents.

## 5. Enzymes Involved in the Degradation of Anthraquinone and Textile Dyes

Enzymatic remediation is a powerful and specific method for the treatment of textile wastewater, particularly for recalcitrant high molecular weight polycyclic structures like the anthraquinone dye RBBR, which requires specific catalytic pathways [96]. RBBR has a stable fused aromatic anthraquinone nucleus, unlike common azo dyes that have unstable azo linkages (–N=N–), and it is resistant to simple reductive cleavage and needs special oxidative pathways to ring opening and detoxification [97].

A systematic assessment of these biocatalytic processes requires a comparative understanding of the catalytic mechanisms of the key enzyme classes, namely azoreductases, laccases, and peroxidases. Azoreductases are enzymes that catalyze the reductive cleavage of azo bonds (−N=N−) in anaerobic or microaerophilic environments with electron transfer from NAD(P)H cofactors [102]. They are efficient for azo dyes, but their catalytic scope is very limited in handling the fused ring structure of anthraquinone dyes such as RBBR, which are mainly based on oxidative transformations. Laccases (benzenediol: oxygen oxidoreductases): Laccases are multi-copper oxidases that catalyze the one-electron oxidation of phenolic and non-phenolic substrates with molecular oxygen as the electron acceptor, yielding water as the byproduct [100]. These produce reactive free-radical intermediates, which induce non-specific ring cleavage, and thus are the main catalysts for the degradation and detoxification of recalcitrant anthraquinone compounds [97]. Peroxidases (manganese peroxidases, lignin peroxidases, and dye-decolorizing peroxidases—DyPs). Peroxidases are enzymes that need hydrogen peroxide (H_2_O_2_) as an oxidant and possess very high redox potentials. They are the only enzymes capable of attacking very stable aromatic matrices through sequential single-electron abstractions and are superior to azoreductases in the oxidative mineralization of RBBR and related polycyclic effluents [96].

This coordinated action of oxidoreductases provides fine chemo-, regio-, and stereoselectivity, enabling rapid degradation of recalcitrant chromophores without disturbing other non-target effluent components.

The mechanistic features of these classes of enzymes give a basis for the understanding of their potential for dye degradation, but their reported treatment performance varies significantly with enzyme source, reaction conditions, and treatment duration. Table 2 summarizes the main data reported in the literature in terms of enzyme source, reaction conditions, treatment time, and decolorization efficiency for the purpose of direct comparison. This comparison provides an integrated assessment of the performance of different enzyme systems and operating conditions for RBBR and related textile dyes, while highlighting the variability among reported studies.

### 5.1. Azoreductase Activity and Its Role in Dye Degradation

The enzymatic catalysts are azoreductases that help break down azo bonds, producing mostly colorless aromatic amine compounds. They are classified as NAD(P)H: flavin oxidoreductase and are composed of flavoproteins [66]. The enzymes are present in either extracellular or intracellular locations of the bacterial cell membrane and are also found in intestinal microbiota [108]. Azoreductase is a key enzyme in the decolorization of textile dyes. They help break down and then cleave azo bonds. This causes the release of aromatic amines. Further breakdown pathways then break down these amines into carbon dioxide and water [22]. Flavin-containing cofactors, when traversing bacterial membranes, face a formidable obstruction, hindering the flow of reduction equivalents from the cytoplasm to sulfonated azo dyes. This obstruction necessitates an alternative route that operates through a non-reductive mechanism, facilitated by cytoplasmic flavin-dependent azoreductases. In Gram-negative bacteria, the electron transport components are located strategically in the outer membrane to interact with the substrate of an azo dye or a redox mediator [109].

Moreover, researchers have demonstrated that consortium-based strategies effectively degrade RBBR by harnessing the metabolic activity of complementary microbes. A consortium of marine-associated bacteria such as *Dietzia maris*, *Bacillus subtilis*, *Marasmiellus* spp. and *Mucor racemosus* decolorized RBBR 86% and detoxified 52% after 7 days. Results of meta-transcriptomic analysis suggested that oxidases, oxygenases and hydrolases participated in the transformation of RBBR, indicating that the synergistic microbial metabolism may make an important contribution to the removal and detoxification of the dye [110]. These results suggested that the microbial consortia may be involved in the treatment of RBBR, and azoreductases were more like a supplement but not an enzyme that could degrade RBBR alone.

However, the extension of these reductive mechanisms to structurally different compounds such as Remazol Brilliant Blue R (RBBR) requires a critical evaluation. Structurally, RBBR is an anthraquinone dye and not an azo dye and as such does not have the characteristic linkage that is targeted by true azoreductases [111]. Classical azoreductases do not catalyze the direct reductive cleavage of the RBBR core as shown by experimental work on purified enzyme systems [112]. Although azoreductases do not directly cleave the anthraquinone structure of RBBR, they may contribute indirectly to dye degradation in mixed microbial consortia [8]. In textile effluents containing both azo and anthraquinone dyes, azoreductase-producing microorganisms can reduce azo bonds in co-existing dyes and transform certain redox-active intermediates [11]. This reductive activity may alter the redox environment, facilitate electron transfer, and generate intermediates that are more amenable to subsequent oxidative attack by laccases or peroxidases [10,12]. In sequential anaerobic–aerobic systems, azoreductase-mediated reduction may occur during the anaerobic phase, whereas oxidative enzymes can further transform residual chromophores and aromatic intermediates under aerobic conditions [13,14]. Therefore, the cooperation between azoreductases and oxidative enzymes in microbial consortia is likely to be complementary rather than direct: azoreductases may broaden the range of degradable dye components and support redox balancing, while laccases and peroxidases primarily initiate the oxidative transformation and ring cleavage of RBBR [12]. Further studies using defined co-cultures, enzyme activity measurements, and intermediate-product identification are needed to confirm the extent and significance of this interaction. Experimental evidence points rather towards oxidative mechanisms controlled mainly by the degradation of RBBR by ligninolytic enzymes like laccases, manganese peroxidases, and versatile peroxidases, which attack the fused anthraquinone rings via free-radical pathways [113]. It is worth noting that crude microbial consortia or intracellular fractions of wide specificity may act on different classes of dyes at the same time. Therefore, RBBR removal cannot be exclusively explained by azoreductase activity, neglecting the crucial oxidative pathways [114].

#### 5.1.1. Enzymatic Mechanics and Structural Configurations of Bacterial Azoreductases

Bacterial azoreductases (EC 1.7.1.6) are a separate family of oxidoreductases that catalyze the reductive cleavage of chromophoric azo bonds using reduced nicotinamide adenine dinucleotide (NADH) or reduced nicotinamide adenine dinucleotide phosphate (NADPH) as electron donors (–N=N–) [83]. These enzymes are structurally organized from simple to complex, defining their catalytic kinetics and remediation efficiency [115]. Flavin-free monomers are structurally simpler but lack redox-active cofactors, making direct electron transfer necessary and thus causing slower catalytic turnover for complex xenobiotic structures. The flavin mononucleotide (FMN)-dependent homotetrameric AzoA from *Enterococcus faecalis* and specialized tetrameric flavoproteins from *Staphylococcus aureus* are flavin-dependent systems that are complex multimeric assemblies, in which both cofactors and substrates are non-covalently coordinated, as shown by size-exclusion chromatography and sodium dodecyl sulfate–polyacrylamide gel electrophoresis (SDS-PAGE).

This architecture in the quaternary structure translates directly to an improved degradation performance: the accurate spatial positioning of the coenzyme, the flavin cofactor, and the target substrate creates a more efficient electron relay, which drastically reduces the activation energy needed for the cleavage of recalcitrant azo bonds [116]. Furthermore, the compact packing of multimeric azoreductases ensures structural stability in fluctuating alkaline and neutral niches, avoiding thermal or chemical denaturation, and allows sustained high-throughput catalytic performance in the treatment of complex industrial dye effluents.

Synthesized by both obligate anaerobes and facultative aerobes, these enzymes catalyze electron cascades that are differentially modulated by molecular oxygen (O_2_). Under anaerobic conditions, azoreduction functions as a terminal electron-accepting process integrated with the microbial electron transport chain (ETC). Cytoplasmic flavoproteins re-oxidize reducing equivalents (NADH or NADPH) to generate reduced flavins (FMN-H_2_) driving two distinct spatial cascades [117].

In addition to the overall structural organization, the architecture of the active site and substrate-binding pocket plays an important role in the catalytic efficiency of bacterial azoreductases. Structural studies show that substrate recognition and catalytic selectivity are mediated by hydrophobic interactions between the FMN isoalloxazine ring and selected residues in the active site. In *Escherichia coli* AzoR for example, mutation of Arg59 dramatically changed the substrate specificity and Vmax for the reduction of p-methyl red illustrating the effect of active-site residues on catalytic properties [89]. Structure-guided engineering of an FMN-dependent azoreductase from *Roseibium* sp. H3510 resulted in a mutant with 3.24-fold increased kcat/Km (catalytic efficiency) and improved thermostability. This improvement was due to the structural stabilization of the FMN-binding pocket and substrate tunnel [55]. These findings provide a structural basis for enzyme engineering and suggest that the catalytic efficiency of azoreductases is determined by the properties of the active-site architecture, the substrate channel, and the stability of the protein.

#### 5.1.2. Biochemical Catabolic Pathways of Azo Dye Mineralization

The chemical structure of synthetic dyes is an important parameter influencing their microbial degradation. The difference in the chromophoric groups in azo dyes and anthraquinone dyes (e.g., RBBR leads to the difference in the enzymatic degradation pathways. Azo dyes have at least one azo (–N=N–) linkage, and anthraquinone dyes possess a fused quinone ring system and lack azo linkages. Their biodegradation mechanisms are thus quite different [118].

**Scheme 2:**
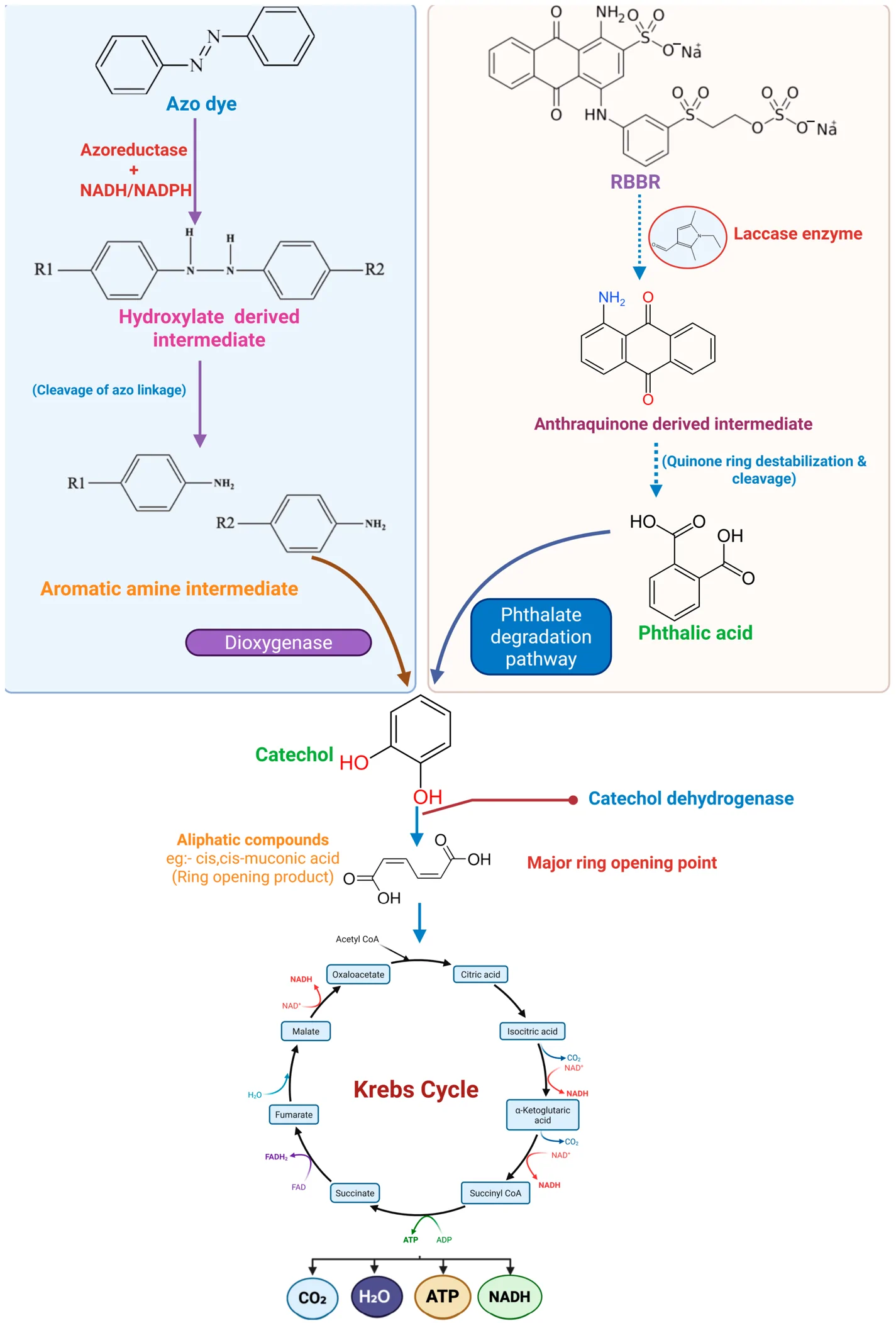
Proposed microbial degradation and mineralization pathways of azo dye and Remazol Brilliant Blue R (RBBR) [104,105,106,107]. Created in BioRender. Pal, D. S. (2026) https://BioRender.com/1s3q83w.

Flavin-dependent enzymes called azoreductases are the first step of degradation of azo dyes, reducing the azo (–N=N–) bond with or as electron donors [119]. The reduction reaction produces unstable hydrazo-derived intermediates that are then cleaved to give colorless aromatic amine intermediates, decolorizing the dye. Dioxygenases, and dehydrogenases further metabolize aromatic amine intermediates, catalyzing hydroxylation, aromatic ring cleavage, and conversion to low-molecular-weight organic acids [26]. These metabolites are taken up by the tricarboxylic acid (TCA) cycle, where they are fully mineralized into carbon dioxide, water, adenosine triphosphate (ATP), and NADH. The proposed microbial degradation and mineralization pathways of azo dye and Remazol Brilliant Blue R (RBBR) are shown in Scheme 2. The degradation of its lignin is initiated by oxidative enzymes such as laccases, lignin peroxidases (LiPs), manganese peroxidases (MnPs), dye-decolorizing peroxidases (DyPs), and monooxygenases [92]. These enzymes oxidize the anthraquinone chromophore, producing hydroxylated intermediates that are cleaved in the quinone ring [120,121]. The subsequent oxidation by oxygenases yields simple organic acids that are fed into the cycle, leading to complete mineralization into ATP, NADH, CO_2_ and H_2_O. The independent reductive and oxidative pathways emphasize the importance of selecting appropriate microorganisms with suitable enzymatic systems for the effective biodegradation of structurally different textile dyes [82].

Decolorization primarily reflects the removal or transformation of the chromophoric structure of RBBR. However, the resulting intermediates may undergo further transformation and mineralization, involving their conversion into CO_2_, H_2_O, and inorganic products, as demonstrated in experimental studies. Importantly, decolorization should not be equated with complete degradation or mineralization of RBBR. Although loss of color indicates transformation of the chromophore, degradation intermediates may remain after treatment. Therefore, mineralization and residual toxicity should be assessed separately using appropriate experimental evidence, such as the identification of degradation products, measurement of total organic carbon or dissolved organic carbon removal, CO_2_ evolution, and toxicity assays.

### 5.2. Laccase-Mediated Degradation of Anthraquinone Colorants

Laccase (EC 1.10.3.2) is a multicopper oxidase enzyme and one of the most studied enzymes for the biodegradation of anthraquinone colorants. This is due to its broad substrate specificity and environmentally benign catalytic mechanism of action. The enzyme catalyzes one-electron oxidations of dye molecules, reducing molecular oxygen to water and generating reactive radicals. These radicals play a role in the cleavage of the stable chromophore of anthraquinone. This leads to effective decolorization and the formation of intermediates with lower molecular weight and less toxicity, which are more susceptible to further microbial mineralization.

Table 3 summarizes the reported functional characteristics and roles of laccase isoforms relevant to RBBR biodegradation, highlighting their potential for efficient dye transformation and supporting their application in enzyme-based treatment systems. The oxidative capacity of laccase can be greatly enhanced in the degradation of recalcitrant anthraquinone dyes by redox mediators such as 2,2-azino-bis (3-ethylbenzothiazoline-6-sulfonic acid) (ABTS) and 1-hydroxybenzotriazole (HBT). Bibi et al. 2025 proved that laccases, especially those from *Pleurotus ostreatus* and *Trametes versicolor*, are highly effective for the treatment of textile effluents containing anthraquinone dyes under mild operating conditions [27].

#### 5.2.1. Catalytic Mechanism and Intramolecular Pathway

**Scheme 3:**
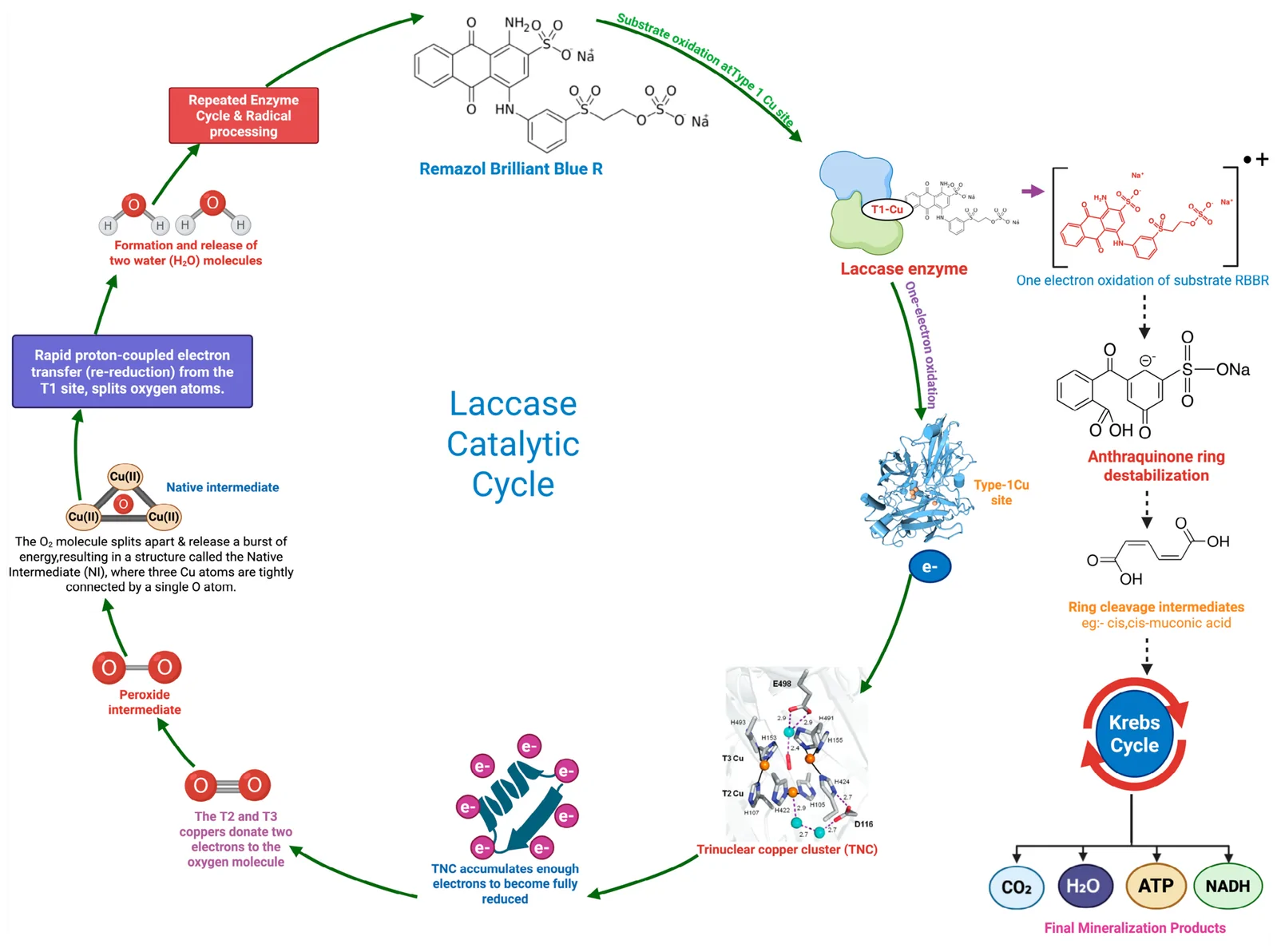
Proposed schematic representation of the laccase catalytic cycle during the biodegradation of Remazol Brilliant Blue R (RBBR). Oxidation of RBBR occurs at the Type 1 (T1) copper center, followed by intramolecular electron transfer to the trinuclear (T2/T3) copper cluster, where molecular oxygen is reduced to water. Repeated oxidation promotes anthraquinone ring cleavage and subsequent mineralization through the tricarboxylic acid (TCA) cycle [122,124]. Created in BioRender. Pal, D. S. (2026) https://BioRender.com/bc3m5cs.

The laccase-mediated catalytic cycle during the structural breakdown of the fused anthraquinone framework of RBBR proceeds via a series of highly coordinated step-by-step electronic transitions. A schematic representation of the proposed laccase catalytic cycle during the biodegradation of Remazol Brilliant Blue R (RBBR) is shown in Scheme 3. The RBBR molecule coordinates are adjacent to the mononuclear Type 1 (T1) copper site in the laccase enzyme. The (T1) site abstracts a single electron from the nucleophilic phenolic or secondary aromatic amine groups flanking the core anthraquinone ring, converting the substrate into a highly reactive radical cation intermediate (Cu^2+^ → Cu^+^) [79]. The captured electron is transferred from the T1 site across a highly conserved internal tripeptide pathway over approximately 3Å to the buried trinuclear cluster, which comprises one Type 2 (T2) and two Type 3 (T3) copper sites [122]. The catalytic reduction of molecular oxygen at the trinuclear copper cluster (TNC) is driven by a highly coordinated sequence of protonation and electron transfer events. Following oxygen binding, the enzyme utilizes conserved acidic amino acid residues, namely aspartate and glutamate, to channel protons into the active site. This proton influx forces the stretched O-O bond to undergo reductive cleavage in a highly exothermic reaction, resulting in the formation of the Native Intermediate (NI). Functioning as the true catalytically relevant state during continuous degradation turnover, the NI is a fully oxidized species characterized by three Cu(II) ions tightly coordinated by a single µ3-oxo bridge. This unique bridged architecture significantly lowers the activation barrier for subsequent electron transfers, facilitating exceptionally rapid proton-coupled electron transfer (PCET) from the Type 1 (T1) copper site. Ultimately, this rapid influx of electrons and protons fully reduces the oxygen atoms, culminating in the release of two H_2_O molecules and resetting the TNC to accept the next oxygen molecule. The trinuclear (T2/T3) cluster coordinates the activation and electron reduction of molecular oxygen (O_2_) to yield two molecules of water (H_2_O), safely completing the enzyme’s catalytic cycle without releasing toxic peroxide intermediates [123].

#### 5.2.2. Radical Stabilization Cascades and Structural Cleavage

The highly unstable free-radical intermediates generated from the RBBR skeleton rapidly propagate through two distinct, competing downstream pathways. The radical state destabilizes the fused ring system, inducing the cleavage of core carbon–carbon (C–C) or carbon–nitrogen (C–N) bonds, structuring the anthraquinone skeleton. This pathway systematically fractures the core chromophore into low-molecular-weight, colorless fragments consisting of simple aromatic amines and phenolic compounds [27]. Alternatively, the transient radicals undergo intermolecular homolytic coupling, creating higher-molecular-weight dimers or complex insoluble polymers that spontaneously precipitate out of the liquid matrix [120]. These primary breakdown fragments undergo further progressive laccase oxidation or subsequent downstream microbial degradation, resulting in complete xenobiotic detoxification. The efficiency of this entire breakdown cascade remains highly contingent upon environmental boundary conditions, including matrix pH, operating temperature, dissolved oxygen levels, the presence of natural mediators, and the specific macrofungal origin of the enzyme system [27,120].

Complementary analytical techniques have been used to provide experimental evidence for the proposed RBBR degradation pathway. FTIR analysis showed that the dye molecule was structurally modified, as the hydroxyl (O–H), amino (N–H), and sulfoxide (S=O) functional groups disappeared or were significantly reduced. Degradation intermediates such as 9,10-anthracenedione-1-amino were identified by GC–MS together with a significant decline in aromatic esters, phenolic compounds, and phthalate derivatives, indicating oxidative transformation of the anthraquinone frame. SDS-PAGE and enzyme assays also confirmed the involvement of lignin peroxidase, manganese peroxidase, and azoreductase. Efficient detoxification of the degradation products was evidenced in phytotoxicity studies [92].

### 5.3. Peroxidase-Mediated Oxidation Catalysis

#### 5.3.1. Biochemical Characteristics and Structural Organization

Peroxidases (EC 1.11.1.X) are a large superfamily of oxidoreductases that are present in plants, fungi, bacteria, and other eukaryotes. These enzymes catalyze the reduction of hydrogen peroxide (H_2_O_2_) or organic peroxides with concurrent oxidation of a wide variety of organic and inorganic substrates. Peroxidases are mostly heme proteins that have a protoporphyrin IX group attached to a ferric iron (Fe^3+^) center. They have a molecular weight between 30 and 150 kDa and generally high redox potentials and can oxidize recalcitrant aromatic pollutants, including phenolic compounds, aromatic amines, lignin-derived compounds, polycyclic aromatic hydrocarbons (PAHs) and anthraquinone dyes like Remazol Brilliant Blue R (RBBR) [54].

The degradation of RBBR has been experimentally reported for several fungal and bacterial peroxidases. For example, the anthraquinone chromophore is efficiently oxidized by *Phanerochaete* chrysosporium-produced lignin peroxidase (LiP) and manganese peroxidase (MnP), which results in significant decolorization and structural degradation of RBBR. Similarly, peroxidase-producing *Stutzerimonas stutzeri* VITKSS2 was reported to decolorize up to 99% of RBBR. FTIR studies confirmed the loss of the major functional groups. GC–MS analysis helped to identify the intermediates of degradation. SDS-PAGE analysis detected the presence of lignin peroxidase and manganese peroxidase, which gave direct experimental proof for peroxidase-mediated RBBR degradation [92].

#### 5.3.2. Catalytic Mechanism

The oxidative degradation of substrates by heme peroxidases proceeds through a characteristic three-stage redox loop driven by a hypervalent iron catalytic center. The catalytic cycle of heme peroxidases begins with activation of the resting ferric enzyme (E, Fe^3+^) by hydrogen peroxide (H_2_O_2_). The binding of H_2_O_2_ leads to heterolytic cleavage, releasing one molecule of water and the formation of Compound I (E–I), a highly reactive oxoferryl porphyrin π-cation radical intermediate. The main oxidizing agent is Compound I, which starts the one-electron oxidation of aromatic substrates, including RBBR, thanks to its high redox potential. This activation step is essential for the oxidation of the substrate and affects the efficiency of the peroxidase-catalyzed biodegradation [125].

E (Fe^3+^) + H_2_O_2_ → E–I (Compound I) + H_2_O

Compound I (E-I) is the first oxidized intermediate of the peroxidase catalytic cycle. It is a strong one-electron oxidant due to its high redox potential. Compound I then reacts with the substrate by accepting one electron from the aromatic π-electron system of RBBR, producing a highly reactive substrate radical cation (RBBR•^+^) and being reduced to Compound II (E–II). The formation of a radical cation destabilizes the anthraquinone chromophore and therefore favors further oxidative transformations such as bond cleavage and structural rearrangement. The electron transfer step is the main oxidative step that initiates the enzymatic degradation of RBBR and allows its subsequent conversion into lower-molecular-weight intermediates through successive catalytic processes.

E–I (Compound I) + RBBR → E–II (Compound II) + RBBR•^+^

After the first substrate oxidation, Compound II (E–II) is reduced by taking a second electron from an additional molecule of RBBR to regenerate the resting ferric peroxidase (E, Fe^3+^) and complete the catalytic cycle. This second one-electron oxidation results in another RBBR radical cation (RBBR•^+^), which is then subjected to additional oxidative reactions, including rearrangement and cleavage of the anthraquinone chromophore. In biodegradation, the native ferric enzyme must be regenerated for peroxidase to achieve continuous catalytic turnover to repeatedly oxidize substrate molecules. The efficient recycling of enzymes is the key for sustainable decolorization, and detoxification of dyes, and this step is critical for the overall catalysis efficiency of peroxidase-mediated RBBR degradation [103].

E–II + RBBR + 2H^+^ → E (Fe^3+^) + RBBR•^+^ + H_2_O

#### 5.3.3. Degradation Cascades and Mineralization

The RBBR radical cation (RBBR•^+^) is successively oxidized following peroxidase oxidation, which leads to the instability of the anthraquinone chromophore, cleavage of the aromatic ring, and formation of lower-molecular-weight metabolites. The proposed degradation pathway has been supported by several analytical techniques, including UV–Vis spectroscopy showing the loss of the characteristic anthraquinone chromophore, FTIR spectroscopy confirming the modification of functional groups, and HPLC, LC–MS, and GC–MS identifying intermediate degradation products formed during enzymatic oxidation [19].

Most recently, Sakthishabarish et al. (2026) [92] experimentally demonstrated the structural transformation of RBBR by FTIR and GC–MS analyses, while SDS-PAGE analysis evidenced the expression of proteins by detecting the corresponding protein bands, and the enzyme activity assay supported their catalytic role in RBBR biodegradation. Furthermore, the phytotoxicity assays indicated a considerable decrease in the toxicity of the breakdown products, demonstrating efficient detoxification. Taken together, our results offer strong experimental evidence for the gradual oxidative breakdown of RBBR to simpler metabolites, which are then integrated into microbial metabolic pathways. However, the full mineralization into carbon dioxide (CO_2_) and water (H_2_O) has not been experimentally proven yet and hence is a hypothesized final step that needs additional validation by biochemical and metabolomic methods [92].

## 6. Laccases and Peroxidases for RBBR Degradation

Complementary oxidative biocatalysts with significant potential for the breakdown of RBBR are laccases and peroxidases (Table 4). Laccases make excellent use of molecular oxygen to oxidize the anthraquinone chromophore, while peroxidases are very oxidative through H_2_O_2_-dependent catalysis. Both enzyme systems can catalyze structural transformation and decolorization of RBBR; however, their performance is dependent on the enzyme source and reaction circumstances. Laccases are especially interesting for sustainable treatment systems since no H_2_O_2_ is required, while peroxidases give excellent oxidative capacity against resistant dye complexes. Their complementary catalytic capabilities consequently allow options to select or combine enzyme systems for efficient removal and detoxification of RBBR [82,122].

## 7. Discussion

Enzymatic biodegradation of Remazol Brilliant Blue R (RBBR) has received much attention as an environmentally sustainable method for the treatment of anthraquinone dye-contaminated wastewater. The catalytic mechanisms, substrate specificities, and degradation efficiencies of azoreductases, laccases, and peroxidases, which have all been explored for dye degradation, differ substantially due to the structural complexity of RBBR. Unlike azo dyes, RBBR does not contain the typical azo (–N=N–) bond but a very stable anthraquinone chromophore, which makes it more susceptible to degradation by oxidative enzymes [82,122].

The three enzyme systems are broad in substrate specificity, and laccases catalyze the one-electron oxidation of the anthraquinone chromophore, leading to radical intermediates destabilizing and degrading the aromatic ring. The potential of laccases to oxidize a large range of phenolic and non-phenolic compounds and to function at mild reaction conditions makes them attractive biocatalysts for RBBR treatment. Similarly, peroxidases, such as lignin peroxidase (LiP), manganese peroxidase (MnP), and versatile peroxidase (VP), have high redox potentials that enable rapid oxidation of the anthraquinone structure by hydrogen-peroxide-dependent catalysis. The laccase- and peroxidase-mediated degradation of RBBR has been well evidenced in several experimental studies [82,92,122] through UV–Vis spectroscopy, FTIR, HPLC, LC–MS, GC–MS, enzyme activity assays, and phytotoxicity analyses, demonstrating the structural transformation, formation of intermediate metabolites, and detoxification.

The main oxidative enzymes involved in RBBR decolorization are laccases and peroxidases. Complementary oxidative processes are operating on the resistant anthraquinone structure of RBBR. In contrast, azoreductases mainly catalyze the reductive cleavage of azo bonds and thus play little direct role in RBBR degradation. However, azoreductases may be indirectly involved in the transformation of dyes in microbial consortia through complementary or sequential metabolic pathways. Thus, laccases and peroxidases are more directly involved in RBBR degradation, but azoreductases may play a secondary role depending on the microbial community composition and environmental conditions.

Conversely, azoreductases have been characterized for the reductive degradation of azo dyes, where cleavage of the azo (–N=N–) linkage is the main catalytic reaction. Thus, their role in RBBR degradation needs to be understood differently. Current studies indicate that bacterial azoreductases with broad substrate specificity might be involved in complementary reduction reactions within multi-enzyme degradation systems, rather than being the primary enzyme responsible for anthraquinone oxidation. Therefore, evidence for azoreductase activity in RBBR degradation is limited in comparison to the extensive experimental validation for laccases and peroxidases, highlighting the need to differentiate the mechanisms reported for azo dyes from those specific to anthraquinone dyes. Enzymatic systems have a high potential to remove dyes, but most of the studies have been performed using pure dyes or artificial wastewater under laboratory conditions. Their performances in complicated textile wastewater have not been well substantiated. Salts, surfactants, auxiliary chemicals, heavy metals, and other organic materials can interfere with enzyme–substrate interactions and reduce enzyme efficacy and stability. At the laboratory scale, enzyme activity is generally maintained under moderate temperature and pH conditions close to the optimum of the specific enzyme. For many laccases, this corresponds to temperatures of approximately 25–40 °C and acidic to near-neutral pH, whereas peroxidase activity also depends on an appropriate hydrogen-peroxide concentration. Extreme pH, high temperature, salinity, surfactants, heavy metals, and excessive oxidant concentrations may reduce activity or cause irreversible enzyme inactivation. Enzyme reuse can be achieved through immobilization on supports such as alginate beads, magnetic nanoparticles, silica, chitosan, or membranes, which facilitates enzyme recovery and repeated batch or continuous operation. Nevertheless, the feasibility of reuse should be assessed by repeated-cycle experiments that measure residual enzyme activity, dye-removal efficiency, and possible enzyme leaching from the support. A key advantage of enzyme-mediated degradation over adsorption- or coagulation-based methods is that enzymes can directly transform the dye chromophore under mild conditions rather than merely transferring the pollutant to another phase [125]. For RBBR, oxidative enzymes can disrupt the conjugated anthraquinone structure and generate lower-molecular-weight intermediates, thereby offering a potentially more sustainable approach when combined with appropriate post-treatment to ensure detoxification and mineralisation. Most dye-treatment studies are based on the measurement of color removal or adsorption rather than enzymatic transformation and detoxification, thus limiting the evaluation of biodegradation. However, the limited availability of pilot- and full-scale studies and insufficient validation in real wastewater conditions hinder the application of enzyme-mediated dye degradation. Although most enzymatic studies have used synthetic dye solutions, enzyme-based technologies have also been evaluated for real azo-dye-containing textile effluents. For example, an engineered laccase from *Cyathus bulleri* was applied in an enzyme membrane reactor to treat combined effluent from a textile mill containing diverse azo dyes. The reactor achieved more than 95% decolorization and approximately 96% reduction in mutagenicity during continuous operation, without requiring a redox mediator [125]. In this configuration, enzymatic degradation was combined with membrane separation: the laccase catalyzed the transformation of dye chromophores, while the membrane retained the biocatalyst and enabled continuous treatment. Such results indicate that enzyme-based processes may be integrated with membrane systems, adsorption, advanced oxidation processes, or conventional biological treatment to improve color removal, detoxification, and mineralization of dye-containing effluents. The combination of enzymatic degradation with other treatment processes can improve the overall performance for complex textile effluents. In a typical treatment train, enzymatic degradation is most appropriately positioned as a secondary or polishing step after preliminary screening, equalization, pH adjustment, or coagulation–flocculation, which can reduce suspended solids and competing contaminants. The enzymatic step can then target residual dissolved dye chromophores that are not effectively removed by separation-based processes. A subsequent adsorption, membrane-filtration, biological, or advanced oxidation step may be used to remove residual color, enzyme residues, degradation intermediates, or remaining organic pollutants. For example, a practical sequence could involve screening and equalization, followed by pH adjustment or coagulation–flocculation, enzymatic degradation, adsorption or membrane filtration, and final biological polishing or advanced oxidation. The optimal order should be selected according to effluent characteristics, enzyme stability, target dye concentration, treatment objective, and cost.

Although biological processes such as activated sludge systems, sequencing batch reactors, membrane bioreactors, constructed wetlands, and fungal or mixed-culture systems have been applied to dye-containing textile effluents at pilot or full scale, their effectiveness for recalcitrant anthraquinone dyes such as RBBR may be reduced by fluctuations in dye concentration, salinity, pH, temperature, hydraulic loading, and the presence of other pollutants. Therefore, further pilot-scale and full-scale investigations are required to assess the technical feasibility, operational stability, treatment cost, scalability, and long-term performance of enzymatic and microbial RBBR-treatment systems under actual textile-wastewater conditions. Future studies should focus on the validation of real effluents, the stability of enzymes, experiments at the reactor scale, and the identification of degradation products and their toxicity [104]. At the laboratory scale, enzyme activity is generally maintained under moderate temperature and pH conditions close to the optimum of the specific enzyme. For many laccases, this corresponds to temperatures of approximately 25–40 °C and acidic to near-neutral pH, whereas peroxidase activity also depends on an appropriate hydrogen-peroxide concentration. Extreme pH, high temperature, salinity, surfactants, heavy metals, and excessive oxidant concentrations may reduce activity or cause irreversible enzyme inactivation. Enzyme reuse can be achieved through immobilization on supports such as alginate beads, magnetic nanoparticles, silica, chitosan, or membranes, which facilitates enzyme recovery and repeated batch or continuous operation. Nevertheless, the feasibility of reuse should be assessed by repeated-cycle experiments that measure residual enzyme activity, dye-removal efficiency, and possible enzyme leaching from the support.

Future research should progress from mechanistic understanding to practical application through coordinated basic and applied-engineering studies. Basic research should focus on identifying RBBR transformation intermediates and complete mineralization pathways using LC–MS/MS and GC–MS, together with toxicity and TOC/DOC analysis to distinguish decolorization from true detoxification. Studies should also evaluate the cooperative roles of laccases, peroxidases, and azoreductases in microbial consortia, particularly under sequential anaerobic–aerobic conditions [13]. Applied research should prioritize robust immobilized laccase and peroxidase systems, protein engineering, and hybrid treatment trains combining enzymatic degradation with adsorption, membrane filtration, biological treatment, or advanced oxidation [49]. Enzyme tolerance to salts, surfactants, auxiliary chemicals, and real textile-effluent matrices should be systematically assessed by measuring residual activity, kinetic parameters, RBBR decolorization, and operational stability [46,47]. Pilot-scale and long-term studies using real effluents are needed to validate treatment performance, intermediate toxicity, reusability, cost, and environmental sustainability [47,49]. These integrated approaches will support the translation of enzymatic RBBR degradation from laboratory proof-of-concept studies to scalable textile-wastewater treatment technologies.

## 8. Conclusions

Enzymatic degradation is a promising and sustainable approach for the treatment of RBBR-contaminated textile wastewater. This review highlights laccases and peroxidases as the principal oxidative enzyme systems for RBBR transformation, while azoreductases may contribute indirectly within microbial consortia and sequential degradation pathways [47]. Enzymatic treatment can disrupt the anthraquinone chromophore and achieve decolorization; however, complete mineralization, intermediate identification, and toxicity assessment are essential to confirm true detoxification. The performance of enzymatic systems is strongly influenced by pH, temperature, dye concentration, enzyme source, immobilization, and the presence of salts, surfactants, auxiliary chemicals, and other pollutants in real textile effluents [46,47]. Further integration of enzyme immobilization, protein engineering, microbial consortia, hybrid treatment systems, and real-effluent validation will be required to develop stable, reusable, and scalable enzymatic technologies for sustainable textile-wastewater treatment [13,49].

## Figures and Tables

**Table 1:** Mechanisms, advantages, and limitations of physicochemical methods for textile-wastewater treatment.

Physicochemical Methods	Mechanism	Application to RBBR Contaminated Water	Advantages	Limitations	References
Coagulation–flocculation	The use of chemicals to bring the negatively charged colloidal particles to a state of equilibrium.	RBBR removal of up to 88.51% with activated carbon-based adsorbents, agriculture-waste adsorbents reported up to 90.82% removal	- A straightforward and economically viable approach. - Full elimination of color.	- Accumulates highly poisonous sludges. - Disposal of garbage is an issue.	[58,59]
Adsorption	Microbial cell walls contain various charged dye substances; they attract and capture them.	RBBR molecules bind to the surface of activated carbon or other adsorbents through physical/chemical interactions.	- A wide variety of inexpensive absorbents are available. - A short and easy way. - Adsorption of various dyes is very high.	- Generation of sludge. - Produces solid waste. - Requires continuous supervision. - High cost of maintenance.	[11,60]
Irradiation	Sterilization of effluents by means of high-energy radiation, such as gamma rays or X-rays.	Photochemical excitation promotes dye transformation, often through reactive species.	- Low turbidity and effective removal of suspended particles - Simple to use, saves time - Efficient and regenerative for subsequent applications	- Needs a lot of dissolved oxygen, a long contact time, and a strong dosage of radiation. - Detrimental to the health of people or other organisms - It removes colors ineffectively.	[55,61,62]
Membrane filtration	A physical separation technique that makes use of membrane filters of varying sizes and characteristics.	Nanofiltration/ultrafiltration systems have been investigated for RBBR removal.	- This strategy is both energy conserving and safe for the environment. - Extremely effective at removing dyes of all types—all natural and chemical-free. - Both heat and chemicals have no effect on it.	- Expensiveness and consequences of membrane fouling. - Ideal for use with low to medium dye concentrations.	[63,64]
Ion exchange	The ability of ion exchange resins to undergo a reversible ion exchange between their liquid and solid phases.	Used for removal of anionic RBBR from aqueous media	- The regeneration process does not result in any loss of absorbency.	- Fails to remove dispersed dyes.	[63,64]
Ozonation	Using ozone gas in an oxidation reaction.	RBBR decolorization/degradation through ozone oxidation	- Gets rid of unpleasant smells and tastes, eliminates organic and inorganic impurities too. - Works across a broad range of pH.	- High energy and capital expenditures. - Byproducts pose health risks and may cause cancer.	[59,65]
Chemical precipitation	Chemical precipitating agents to convert soluble metal compounds to insoluble particles.	Conversion of dissolved dye into an insoluble precipitate	- Easy to implement with minimal initial investment.	- Waste disposal, significant maintenance costs, and massive sludge production. - The dispersion dyes do not benefit from it.	[59,66]
Photo catalysis	Making use of light energy to break down organic contaminants.	A light-activated catalyst generates reactive oxygen species that oxidize RBBR.	- Cost effective. - Safe for the planet. - Extremely stable chemically and physically.	- Minimal effectiveness. - Relies heavily on sunshine.	[59,67]
Oxidization	Reduction of pollutants by oxidation from their complicated structures to their simpler, shorter-chained forms.	Fenton, electrochemical, and other oxidation processes have been reported for RBBR treatment.	- Successful degradation and degeneration of oxidizing agent.	- Highly depends on pH and oxidizing agent. - Costlier and commercially unattractive method.	[68,69]

**Table 2:** Comparative summary of enzymatic degradation of RBBR, including enzyme source, initial RBBR concentration, key reactions, decolorization efficiency, treatment time, and corresponding references.

Enzyme	Bacteria Involved	RBBR Concentration	Key Reaction Condition	RBBR Decolorization	Treatment Time	Reference
Laccase	*Ganoderma lucidum*	200 mg/L	pH 4.0, 35 °C, with 0.1 mM vanilin as the redox mediator	98.7%	30 min	[103]
Immobilized spore laccase	*Bacillus subtilis* D1023	100 mg/L	pH 7.0, 37 °C	94.54%	50 h	[104]
Laccase + DyP	*Pleurotus ostreatus*	500 mg/L	Cell-free extracellular extract	98.0%	34 h	[105]
MnP	*Trametes polyzona*	25 mg/L	pH 4.5, 30 °C H_2_O_2_ + Mn^2+^ media for free	100%	30 min	[106]
MnP and laccase	*Irpex lacteus*	150 mg/L	28 °C	100%	144 h	[107]
FMN-dependent NADH-azoreductase-associated system	*Klebsiella* sp. KL-1	100 mg/L	Xylose co-metabolism	80%	48 h	[107]

**Table 3:** Functional significance of laccase isoforms in the biodegradation of RBBR.

Laccase Isoform	Functional Significance	Role in RBBR Degradation	Reference(s)
POXC	Predominant extracellular laccase is expressed under diverse culture conditions and exhibits high catalytic efficiency and broad substrate specificity.	Efficient oxidative decolorization of RBBR exhibits synergistic enhancement when combined with POXA3a or POXA3b.	[122]
POXA1w	Accessory laccase isoform induced under specific environmental conditions; contributes to extracellular oxidative capacity.	Limited direct evidence for RBBR degradation; likely contributes to the overall ligninolytic enzyme system.	[122]
POXA1b	Accessory isoform with distinct catalytic properties and differential gene regulation under dye-induced stress.	Supports oxidation of aromatic compounds and may contribute indirectly to dye degradation.	
POXA3a	Isoform exhibiting high sequence similarity to POXA3b but distinct physicochemical characteristics.	Independently decolorizes RBBR and displays enhanced degradation efficiency in combination with POXC.	[122]
POXA3b	Structurally like POXA3a but differs in chromatographic behavior, suggesting functional diversity.	Comparable RBBR decolorization activity to POXA3a; synergistic interaction with POXC enhances dye degradation.	

**Table 4:** Comparative roles and degradation potential of laccases and peroxidases in RBBR [82].

Feature	Laccases	Peroxidases
Enzyme class	Multicopper oxidases	Heme-containing oxidoreductases
Catalytic mechanism	One-electron oxidation of substrates coupled with reduction of O_2_ to H_2_O	H_2_O_2_-dependent oxidation through high-valent enzyme intermediates
Primary co-substrate	Molecular O_2_	H_2_O_2_
Cofactor	Multiple copper centers	Heme
Action on RBBR	Oxidative transformation of the anthraquinone chromophore and generation of reactive intermediates	Strong oxidative transformation of recalcitrant anthraquinone structures
Major advantage	Does not require external H_2_O_2_; suitable for relatively mild oxidative conditions	High redox potential and broad oxidative capacity toward recalcitrant substrates
Major limitation	Some non-phenolics or highly recalcitrant substrates may require mediators	Excess H_2_O_2_ can cause enzyme inactivation
Operational consideration	Mediator use, pH, temperature, and enzyme stability influence performance	H_2_O_2_ concentration and reaction conditions must be carefully controlled
Relevance to RBBR treatment	Demonstrated potential for RBBR decolorization and oxidative transformation	Demonstrated potential for degradation of recalcitrant textile dyes, including anthraquinone dyes
Overall application potential	Attractive for sustainable and mediator-free systems where suitable substrates are available	Attractive where high oxidative potential is required and H_2_O_2_ can be controlled

## Data Availability

No new data were created or analyzed in this study. Data sharing is not applicable to this article.

## Abbreviations

The following abbreviations are used in this manuscript:

RBBR	Remazol Brilliant Blue R
FMN	Flavin Mononucleotide
SDS-PAGE	Sodium Dodecyl Sulfate–Polyacrylamide
ETC	Electron Transport Chain
ABTS	Azino-Bis (3-Ethylbenzothiazoline-6-Sulfonic Acid)
HBT	1-Hydroxybenzotriazole
T1	Type 1
T2	Type 2
T3	Type 3
FTIR	Fourier Transform Infrared Spectroscopy
PAHs	Polycyclic Aromatic Hydrocarbons
HPLC	High-Performance Liquid Chromatography
LC-MS	Polycyclic Aromatic Hydrocarbons
IET	Intramolecular Electron Transfer
VP	Versatile Peroxidase
MLD	Million Liters Per Day
ETPs	Effluent Treatment Plants
TDS	Total Dissolved Solids
